# In Situ Monitoring of Anodic Acidification Process Using 3D μ-XCT Method

**DOI:** 10.3390/ma17225662

**Published:** 2024-11-20

**Authors:** Chaoqun Zeng, Shanshan Qin, Zhijun Deng, Miaochang Zhu

**Affiliations:** 1 School of Automotive and Transportation Engineering, Shenzhen Polytechnic University, Shenzhen 518055, China; zengchaoqun@szpu.edu.cn (C.Z.); dengzhijun@szpu.edu.cn (Z.D.); 2 School of Construction Engineering, Shenzhen Polytechnic University, Shenzhen 518055, China; qinshanshan@szpu.edu.cn; 3 School of Civil and Transportation Engineering, Guangdong University of Technology, Guangzhou 510006, China

**Keywords:** anode acidification, ICCP system, bonding strength, durability

## Abstract

Debonding of the primary anode caused by anodic acidification is one of the major failure modes of the impressed current cathodic protection (ICCP) system in reinforced concrete structures. This study used 3D micro X-ray computed tomography (μ-XCT) to monitor the in situ evolution of the anodic acidification-affected zone. Samples were scanned after 0 to 40 days of the accelerated anodic acidification test. The anodic acidification-affected zone was identified in μ-XCT images using the gray level segmentation method. The total volume of this zone was measured using the 3D reconstruction method. It was found that detailed 3D information can be extracted using the 3D reconstruction method. The spatial heterogeneity was analyzed using this reconstructed volume information. The Faraday efficiency was calculated and found to increase after 20 days of operation. It was also found that the affected zone was proportional to the input electrical energy. The proposed model is useful for estimating the durability of an ICCP system.

## 1. Introduction

Impressed current cathodic protection (ICCP) is an effective method for preventing corrosion of the steel bars embedded in reinforced concrete structures, especially for structures exposed to a high chloride environment [1,2,3,4,5]. The principle of ICCP is to apply a uniform electric current to lower the chemical activity of the steel bars, where they serve as a cathode. Long-term application of ICCP on bridges and ports shows that it could maintain the integrity of RC structures for up to 50 years [2,6,7,8]. To close the electrical circuit, an external anode material should be attached to the surface of the targeted structures. In general, the anode system is composed of two separate substances: a primary anode and a secondary anode [9]. In most cases, the primary anode is a highly conductive and corrosion-resistant material, such as graphite or titanium mesh. The secondary anode, which also serves as a binder, is generally made of cementitious mortar [10,11,12]. The role of the secondary anode is to distribute the electrical current and keep the primary anode attached to the targeted structures.

The loss of the binding ability of the secondary anode due to anodic acidification is a major cause of the ultimate failure of the ICCP system [1]. Anodic acidification is caused by continuous neutralization of the hydroxide ions around the interface between the primary and secondary anodes:

(1) $$4OH^{-}\to 2H_{2}O+O_{2}+4e^{-}$$

The resultant effect is a decrease in the alkalinity of the pore solution in the interface between the primary and the secondary anodes [13,14]. This leads to the dissolution of calcium hydroxide in the cement paste contained in the secondary anode, which, in turn, decreases both the electrical conductivity and the bond strength of the secondary anode. The ultimate effect is the complete debonding of the primary anode from the concrete substrate, leading to the failure of the ICCP system.

Although the mechanism of anodic acidification is well known, no quantitative study has been conducted on the volume growth of the anodic acidification-affected zone. Polder et al. first provided a rough estimation of the anodic acidification-affected zone. They concluded that the loss of the bonding ability of the anode interface occurs when the depth of the affected zone exceeds 50 μm [1]. This estimation is rather empirical and needs to be further confirmed. Zhang et al. conducted a series of experimental investigations on the electrical transmission ability of the anode interface [15]. They showed that the anodic acidification greatly affects the electrical transmission ability of the primary/secondary anode interface. They also found that the depth of the anodic acidification-affected zone is not uniform around the anode/mortar interface [14]. These findings showed that anodic acidification is a complex phenomenon, and further investigations are needed to provide a quantitative estimation of the evolution of the anodic acidification-affected zone.

On the other hand, X-ray computed tomography (XCT) allows in situ, non-destructive monitoring of the internal microstructure of materials. These prominent characteristics and advantages have attracted tentative cement and concrete research applications [16,17]. These include identifying internal material components to determine the cement hydration and hardening process, developing micropore structure, and migration into the concrete material, etc.

This article proposes a quantitative study of the anodic acidification process using the 3D μ-XCT method. Specimens of mixed metal oxide (MMO)-coated titanium bars served as the primary anode, with cement paste as the secondary anode. Accelerated acidification tests were conducted in a simulated pore solution. In situ monitoring of the anodic acidification-affected zone was conducted using the 3D μ-XCT method. After the accelerated acidification tests, the sample was also characterized using various microscopic methods, including optical microscopy, scanning electron microscopy (SEM), and energy dispersive spectroscopy (EDS). It was found that the primary anode showed no sign of corrosion, and a clear front existed between the anodic acidification-affected zone and the sound zone in the secondary anode. Faraday laws could characterize the total affected volume. Finally, a good linear relationship was found between the input electrical energy and the volume of the anodic acidification-affected zone.

## 2. Materials and Methods

### 2.1. Specimen Preparation

**Figure 1:**
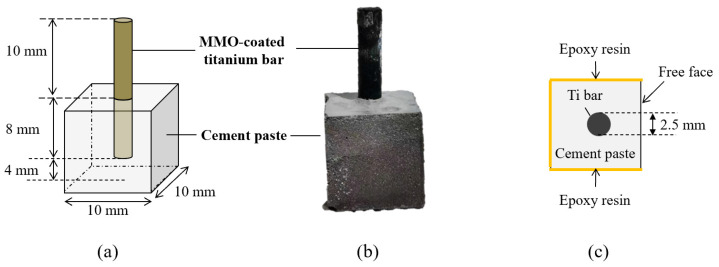
Specimen preparation for accelerated acidification test: (**a**) side view; (**b**) real specimen photo; (**c**) front view.

The anode body was composed of a titanium bar coated with mixed metal oxide (MMO, provided by Zhongtai Metal Materials Co., Ltd., Shanxi, China), as shown in Figure 1. The coating on the titanium substrate was a mixture of iridium and tantalum metal oxide (IO_2_ and Ta_2_O_5_), suitable for a high thermal environment, stable crystal structure, catalysis, and good conductivity. The diameter of the anode bar was 2.5 mm, embedded in cement paste. The dimensions of the specimens are illustrated in Figure 1.

The secondary anode was fabricated by mixing ordinary Portland cement (OPC P II 42.5, provided by China Resources Cement Holdings Ltd., Guangzhou, China) powder and water. The composition of the cement powder is shown in Table 1. The geometric dimensions of the sample are also indicated in Figure 1. The water-to-cement ratio was chosen to be 0.5. The cast sample was cured at room temperature for 28 days before applying the current density. To simulate the ICCP application’s environment, five of the cubes’ faces were sealed with electrically insulating epoxy resin, and only one face was electrically conducting.

### 2.2. Accelerated Acidification Test

**Figure 2:**
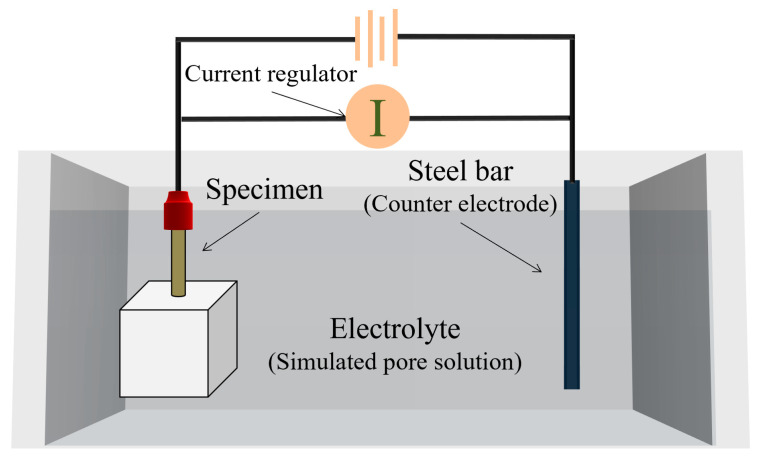
Experimental setup of the accelerated acidification test.

An accelerated acidification test was designed to obtain a sufficient degree of anodic acidification in a relatively short period. The experimental setup of the accelerated acidification test is shown in Figure 2. The accelerated acidification test was conducted in a simulated pore solution. The composition of the simulated pore solution was chosen according to NACE TM0294-2016 [18], as indicated in Table 2. The concentrations of Ca(OH)_2_, NaOH, and KOH were 0.002 mol/L, 0.06 mol/L, and 0.18 mol/L, respectively; the pH of the simulated concrete pore solution was 13.29. To simulate a chloride-contaminated environment, 3.2%wt. KCl was added. The MMO-coated titanium bar was linked to the positive pole of a DC power drive (galvanostat, CorrTest CS1002, Wuhan, China). The counter electrode is made of a stainless-steel plate. The input electrical current density was chosen by considering both the total duration of the accelerated test and the security reason. The electrical current should not be too high, or the driven voltage will be unsafe (>36 V) at the end of the test. It should not be too small; otherwise, the duration of the test will be too long. The authors of [14] chose 2 A/m^2^ and 6 A/m^2^ as the input current densities. Thus, the anodic current density was set to 4.07 A/m^2^ in this experiment, which corresponded to 0.32 mA during the test. Such current density can cause rapid degradation of the anode interface, thus accelerating the anodic acidification effect. The duration of the accelerated acidification test ranged from 0 to 40 days. The driven voltage of the system was monitored during the whole accelerated acidification test duration. Q235 low-carbon construction steel (Shaoguan Iron and Steel Plant, Shaoguan, China) with a diameter of 8.00 mm was applied as the primary anode and cathode in the simulated ICCP system.

### 2.3. Three-Dimensional Inspection of the Sample After the Acceleration Test

**Figure 3:**
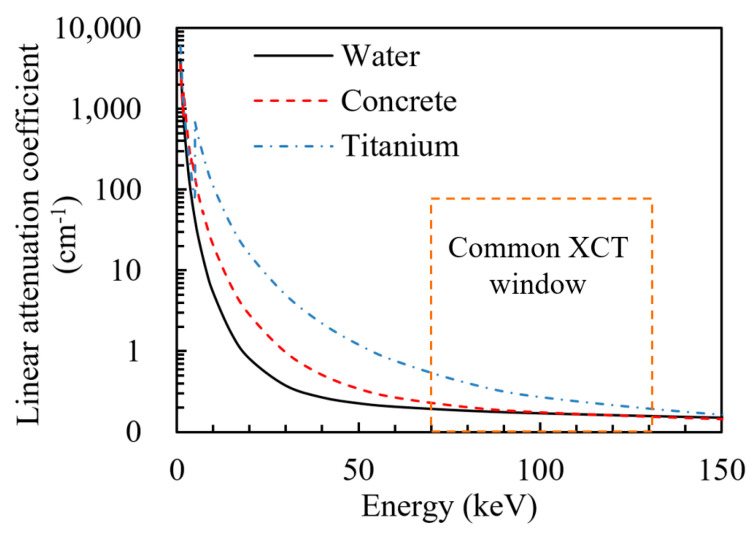
Standard linear attenuation coefficient of the component in our samples according to the National Institute of Standards and Technology [19].

X-ray computed tomography is a non-destructive method that can provide a 3D inside view of porous material. High-energy X-rays can penetrate dense material, including concrete and metal, but gradually lose their intensity within the depth of the material. This phenomenon is called X-ray attenuation. Indeed, for an incident X-ray beam of intensity *I*_0_, the transmitted density of the X-ray beam through a material is an exponential decay function of its thickness x according to the Beer–Lambert law [17]:

(2) $$I=I_{0}e^{-\mu (p,\;Z,\;E)x},$$

where *μ* is the linear attenuation coefficient of the material, *p* is the mass density, *Z* is its atomic number, and *E* is its energy. The principle of the X-ray computed tomography distinguishes different materials based on the difference in the X-ray linear attenuation coefficient. For materials with a low mass density, such as air and water, the linear attenuation coefficient is low. Therefore, high-energy X-rays can pass through these materials with low loss of intensity. The linear attenuation coefficient is relatively high for dense materials such as iron and titanium. The intensity received in the detector is significantly lower. Figure 3 shows the standard linear attenuation coefficient for elements in the prepared samples. It can be seen from Figure 3 that the linear attenuation coefficient is very different in the range of 20 keV to 80 keV [16]. Therefore, for incident X-ray beams with energy between 20 keV and 80 keV, the different components of the material can be distinguished using their linear attenuation coefficient.

For a heterogeneous composite material that contains materials of n different attenuation coefficients, the following equation can calculate the transmitted intensity:

(3) $$ln\frac{I_{0}}{I_{n}}=(\mu_{1}+\mu_{2}+\cdots +\mu_{n})x,$$

where *I_n_* is the intensity received in the detector, and *μ*_1_, *μ*_2_, and *μ_n_* are the linear attenuation coefficients of the respective material.

Finally, 3D object reconstruction can be performed based on Equations (2) and (3). For each sample, 1024 images of X-ray projection from different angles are collected in the database. The projection is defined according to a specific angular position of the object to be reconstructed. Each projection is a 2D matrix with linear attenuation coefficients (pixels). The XCT projection is converted to 8-bit numerical images with 256 (0–255) gray scales. Three-dimensional reconstruction of the samples is computed using a “back-projection” algorithm from the projection images. Based on the variation in pixel intensity with CT attenuation value (Hounsfield units-HU), the structural and material characteristics can be reconstructed and displayed in 3D imaging software (ImageJ, Fiji, https://imagej.net/, accessed on 8 July 2024).

**Figure 4:**
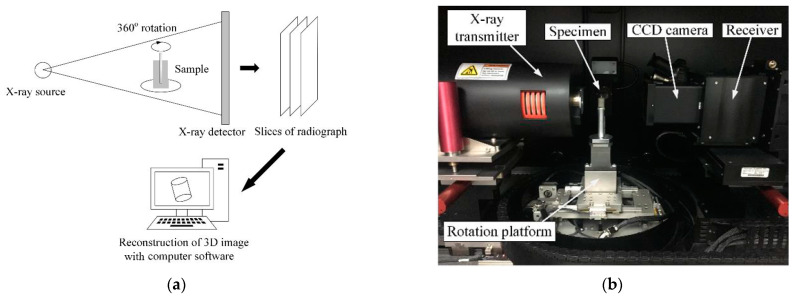
Inspection of the anodic acidification-affected zone using 3D μ-XCT technique after accelerated acidification test: (**a**) principle of X-ray computed tomography; (**b**) photo of the μ-XCT device.

After the accelerated acidification test, the Micro XCT-400, produced by Xradia (produced by Zeiss Group, Oberkochen, Germany), was employed to obtain a 3D view of the samples. The system’s main component includes an X-ray emitter, a rotation stage that allows 360-degree in-plane sample rotation, and a charge-coupled device (CCD) camera, as shown in Figure 4. The X-ray source excitation voltage and current were set to 85 kV and 117 μA, respectively. The 360-degree rotation allows 3D reconstruction of the material phase with the inverse FTT algorithm. Here, the μ-XCT scan was used to provide a 3D view of the effect of the anodic polarization on the material loss in the anode interface, as shown in Figure 4.

Moreover, the optical magnification factor was set to 0.39. It is also worth noting that the pixel size scanned using the μ-XCT technique is dependent on the dimensions of the specimen. The corresponding pixel size down to 20.8 μm was adopted for scanning the whole specimen configuration.

### 2.4. Inspection of the Anodic Acidification-Affected Zone with Optical Microscope and Scanning Electron Microscope

After 40 days of the accelerated acidification test, the sample was cut in the middle, in a direction parallel to the cross section. The height of the cutting was 6 mm. Firstly, the cut surface was observed under an optical microscope. A high-power microscope (Olympus SZX10, Shinjuku, Japan) with a magnification range of 20× to 50× was employed to observe the morphology of the anode interface under a natural light source. Secondly, the surface morphology of the anodic acidification-affected zone was also analyzed with an environmental scanning electron microscope (SEM, Quanta FEG-250, produced by FEI, Hillsboro, OR, USA). The Quanta FEG-250 SEM instrument is an environmental scanning electron microscope used for high-resolution imaging and composition analysis with energy-dispersive X-ray microanalysis (EDS). The FEG column in Quanta 250 allows beam deceleration, which permits a resolution of 1.4 nm even at 1 kV electron landing voltage. For general microscopic morphology observation, the secondary mode (SE) of the scanning electron microscope was adopted, with an acceleration voltage of 10.0 kV, a working distance of 9.5 to 10.5 mm, and a corresponding magnification of 3000 times. In this study, the local Calcium-Si ratio statistics of the external anode were tested using the X-ray energy spectrum.

## 3. Results

### 3.1. Evolution of the Driven Voltage

**Figure 5:**
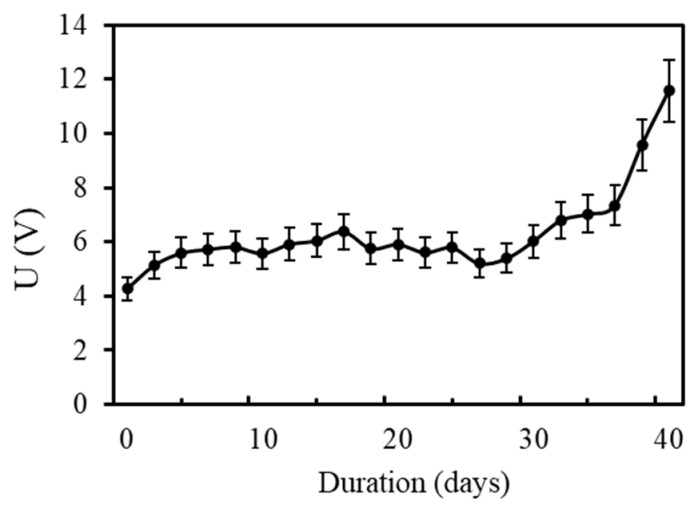
Evolution of the driven voltage during the accelerated acidification test.

The evolution of the driven voltage during the accelerated ICCP test is shown in Figure 5. The figure shows that the driven voltage increases slowly from 4.31 V to 5.93 V during the first ten days of the accelerated test and remains stable around 6 V from day 6 to day 30. A rapid increase in the driven voltage is observed after 36 days of the accelerated test. The voltage increases from 7.48 V to 12 V. This significant increase in the driven voltage is usually associated with anodic acidification of the cement paste around the primary/secondary anode interface. Zhang et al. conducted a similar experiment, and a rapid increase was observed after 30 days of the test with 4 A/m^2^ and after 75 days of the test with 2 A/m^2^ [15]. It is commonly believed that the electrical resistance of a cementitious material depends mainly on the capillary pores inside it. The pH value of the micropores inside the cement paste is around 12.9, which is mainly due to the hydroxide ions released by Ca(OH)_2_. When anodic current is applied to the anode interface, the electric current might cause dissolution of the Ca(OH)_2_ and consume the hydroxide ions, which decreases the pH of the pore solution around the anode interface. Therefore, the anode acidification decreases the amount of free hydroxide ions, which is the main electrical conduction factor. The rapid increase in the driven voltage after 36 days of operation time indicates that the hydroxide ions are almost depleted at this moment. Finally, when the hydroxide ions are totally consumed, the pH of the pore solution decreases to 7, which means that the electrical conductivity of the pore solution rapidly increases to the conductivity of pure water.

Many practical engineers use the point at which there is a significant increase in the driven voltage as a sign of the misfunctioning of the ICCP system. It can be seen from Figure 5 that the operation time is only about 30 days, which is in agreement with the results obtained by Zhang et al. [15].

### 3.2. Growth Rate of the Anodic Acidification-Affected Zone

**Figure 6:**
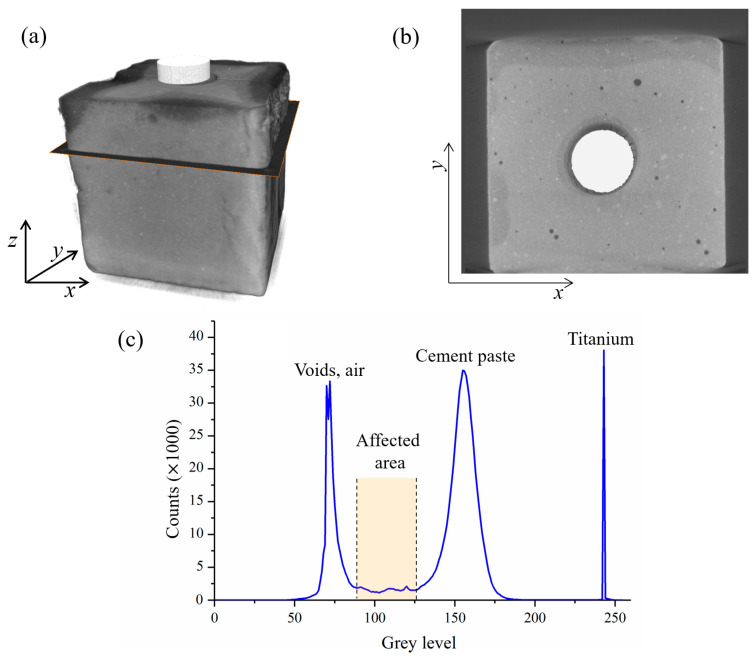
Phase identification process of the anodic acidification-affected zone: (**a**) 3D construction of the samples; (**b**) 2D section view of the scanned image; (**c**) histogram of the gray level clearly showing the gray level of the affected zone.

The sample was scanned using the μ-XCT device after 0, 10, 20, 30, and 40 days of the accelerated test. The reconstructed 3D sample is shown in Figure 6a, while a typical scan result is shown in Figure 6b. The raw image of the scan result is a 1012 × 1024-pixel gray image. The spatial resolution of each pixel is 20.8 μm. The gray level of each pixel is proportional to the linear attenuation coefficient of the material. A high linear attenuation coefficient of the material can be identified using a high gray level pixel in the recorded images. Hence, the general shape of the MMO anode in the center and the surrounding cement paste can be identified in the scanned image.

**Figure 7:**
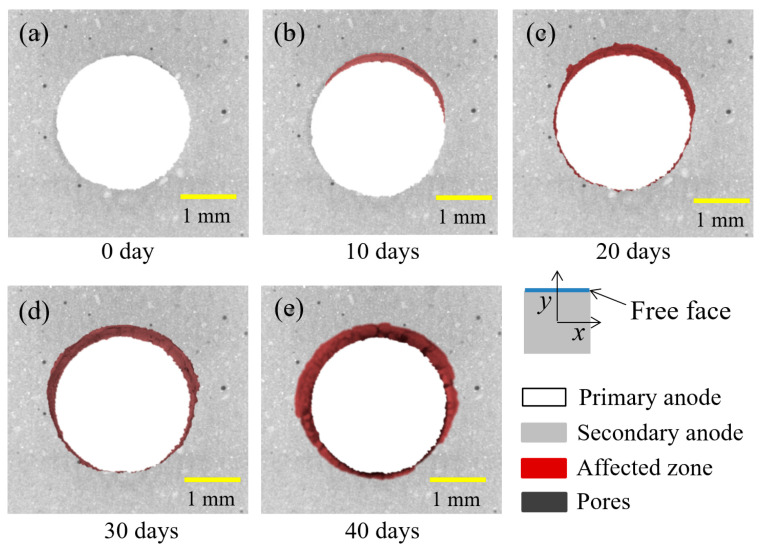
Results of the identification process. The identified anodic acidification-affected zone is shown in dark red. The duration of the accelerated ICCP test increased from 0 to 40 days for (**a**–**e**) with increasing order.

To distinguish the anodic acidification-affected zone in the raw image, an analysis of the gray level based on the histogram was performed. Three horizontal line peaks were drawn in three typical zones. The upper line represents the space occupied only by gas, while the lower line is occupied by pores and cement paste. The line in the middle includes an anode, cement paste, and anodic acidification-affected zone. Figure 6c displays the gray level along the x-direction in these three lines. It can be seen from Figure 6c that the gray level indicates four different phases. The corresponding material phase and the interval of the gray level are shown in Table 3. Hence, using this method, the different phases of the materials, especially the anodic acidification-affected zone, can be identified in the scanned images. The results of the identification are shown in Figure 7.

### 3.3. Three-Dimensional Reconstruction of the Anodic Acidification-Affected Zone

**Figure 8:**
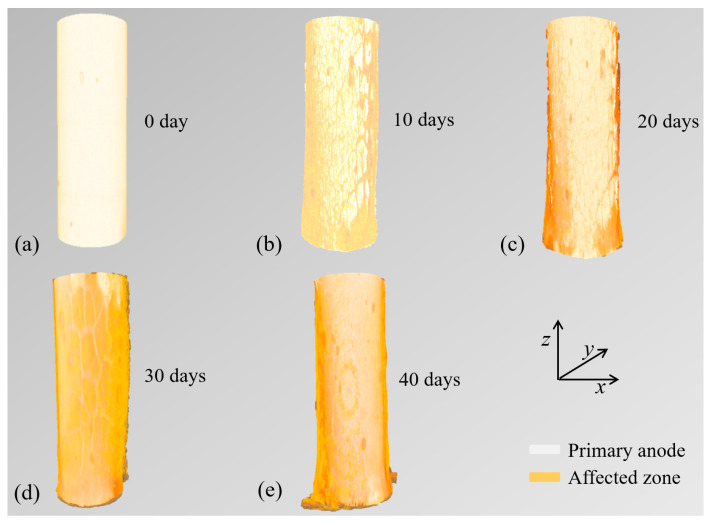
A 3D reconstruction of the anodic acidification-affected zone. The duration of the test from (**a**–**e**) is 0 to 40 days with increasing order.

To further characterize the geometric information from the raw images, such as volume, surface topography, and perimeter, the 3D structure of the samples was reconstructed with the target materials separated based on various color codes, as shown in Figure 8. The main body of the primary anode is shown in white, and the anodic acidification-affected zone is shown in orange. The shape and the volume of the anodic acidification-affected zone were clearly and accurately reconstructed. It can also be seen from Figure 8 that the volume of the anodic acidification-affected zone grew progressively during the accelerated ICCP test.

Furthermore, the volume of the anodic acidification-affected zone can be calculated as:

(4) $$V=N_{vox}Z_{vox},$$

where *V* is the volume of the anodic acidification-affected zone, *N_vox_* is the number of the voxel in the label space, and *Z_vox_* is the spatial voxel size (mm^3^) of the image. To estimate the possible corrosion of the primary anode, its volume was also calculated using the above equation. The calculated volume of the anodic acidification-affected zone and the volume of the primary anode are listed in Table 4. It can be seen from this table that the volume of the primary anode was nearly identical during the accelerated ICCP test, which further confirms that the primary anode has excellent corrosion resistance. By contrast, the volume of the anodic acidification-affected zone grew quickly with the duration of the test. The volume of the anodic acidification-affected zone reached 19.25 mm^3^ at the end of the 40 days of the accelerated ICCP test, which corresponds to an average depth of the affected zone of 0.24 mm. Finally, the results at 40 days show that the total interface was affected by the anodic acidification, which can explain the rapid increase in the driven voltage shown in Figure 5.

### 3.4. Optical and SEM Verification

**Figure 9:**
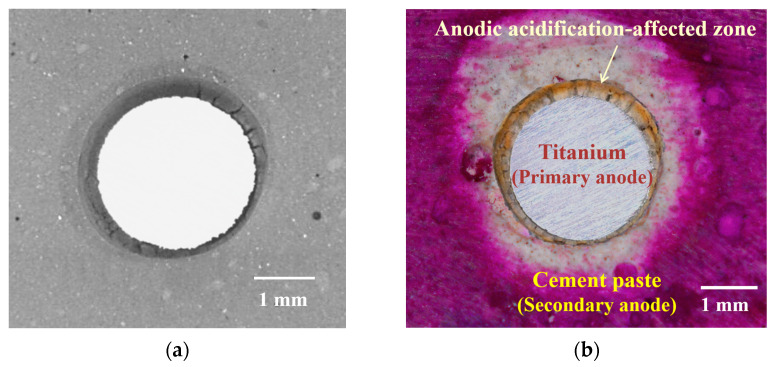
Comparison of the μ-XCT image and optical analysis of the sample at the end of the accelerated ICCP test: (**a**) μ-XCT image; (**b**) optical image with a phenolphthalein pH indicator spray on the surface of the sample.

An optical analysis of the anodic acidification-affected zone was performed at the end of the μ-XCT monitoring to verify the μ-XCT results. The anodic acidification induced calcium leaching inside the cement paste. It was observed that this calcium leaching caused an obvious change in the color of the material. A typical comparison between the μ-XCT image and optical analysis results is shown in Figure 9. The optical image with a phenolphthalein pH indicator showed a clear sign of anodic acidification around the primary anode. A clear color distinction can also be observed between the three material phases, i.e., the primary anode (white metal), the secondary anode (light gray), and the anodic acidification-affected zone. The anodic acidification-affected zone was found in the vicinity of the primary/secondary interface. It can be seen from Figure 9b that the outline of the titanium body was clear and showed no sign of corrosion. The diameter of the primary anode was identical to the original value. This further confirms that the yellow zone is the anodic acidification-affected zone. From Figure 9b, it can also be seen that a clear frontier existed between the anodic acidification-affected zone (yellow) and the intact zone (light gray). This observation confirms that the anodic acidification caused a structural change in the substance in the cement paste, which agrees well with the μ-XCT results.

**Figure 10:**
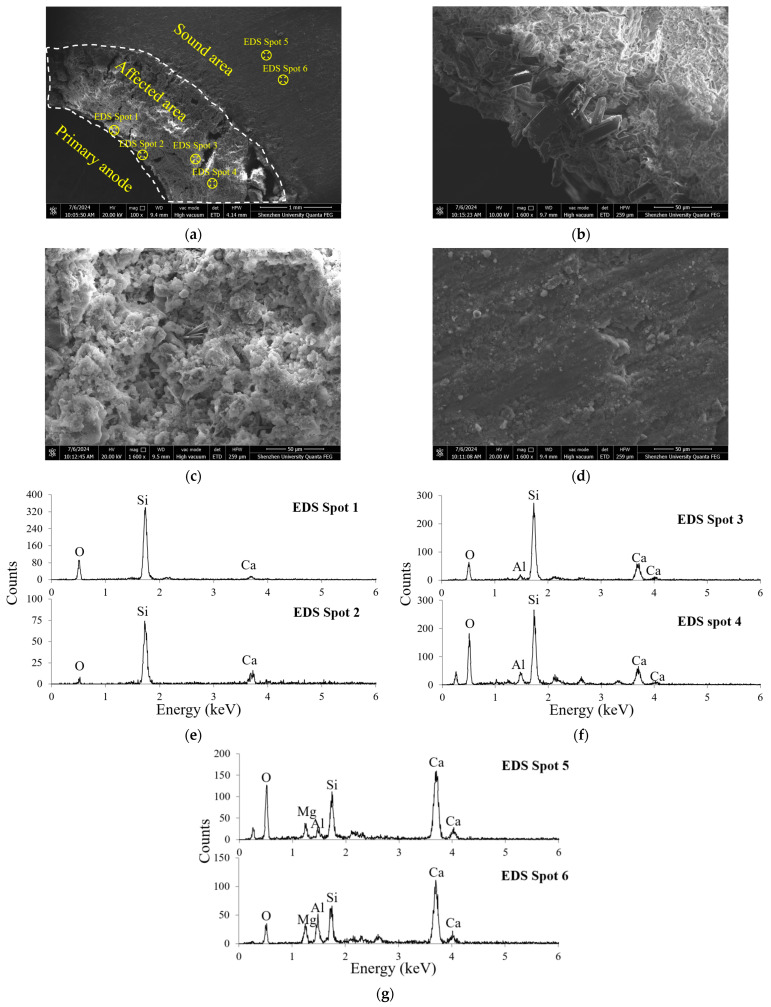
SEM and EDS results for the sample after 40 days of accelerated anode acidification test: (**a**) SEM view at low magnification; (**b**–**d**) increased magnification of EDS spot 1, 3, 5, respectively; (**e**–**g**) EDS results for three points selected from the sample surface.

SEM analysis and EDS analysis were also performed to confirm the structural change in the cement paste after anodic polarization. The results are shown in Figure 10, from which it can be seen that the affected zone shows a more porous structure compared to the unaffected zone. Compared with those in the zones not eroded by acidification, the hydration products of the zones with acidification are sparse and granular. This indicates that due to the loss of calcium ions caused by anodic acidification, calcium hydroxide in the hydration products of the anodic interface region fully participated in the reaction, leading to a significant increase in the pore size of hydration products and a thinning of substances. By contrast, it was found that the structure of anodic acidification products was slightly denser in the unaffected zone.

Furthermore, the EDS spectrum shows a significant drop in calcium concentration around the affected zone. In point A, near the primary anode, there is practically no calcium element in the EDS spectrum. This is because nearly all the Ca(OH_)2_ was consumed during the anodic acidification process. However, point C, the unaffected zone, shows a strong peak in calcium element, indicating an abundant presence of Ca(OH)_2_. In quantitative analyses, the calcium-to-silicon (Ca/Si) ratio has commonly been used to characterize the degree of calcium leaching during the anodic acidification process. The Ca/Si ratios for points A, B, and C are 0.029, 0.21, and 1.52, respectively. These results further confirm that anodic acidification caused the calcium leaching in the secondary anode near the primary/secondary anode interface, and the zone in yellow indicated in Figure 9 is the product of anodic acidification.

## 4. Discussion

### 4.1. Calculation of the Faraday Current Efficiency

It can be seen from the observation of the morphology described in Section 3.2 and Section 3.3 that an obvious zone is affected by acidification at the anode interface. This zone is distinguished from the unaffected zone in terms of color and microstructure due to the loss of calcium ions caused by the electrochemical reaction of the anode current. Therefore, the volume of the anode interface affected by acidification can be evaluated by using the theoretical value calculation and then compared with the experimental results. The theoretical calculation is based on Faraday’s law. Assuming that the electrochemical reaction rate of the anode region is constant, it can be inferred from Faraday’s law that the mass of the anode interface region is related to the input electrical charge as follows:

(5) $$m_{Ca}=k\frac{MIt}{nF},$$

where *k* represents the Faraday current efficiency factor of reaction (1), *M* represents the atomic mass of calcium oxide, *I* represents the input electrical current, *t* represents the duration of the test (in seconds), n represents the degree of ionization of the calcium atom (=2), and *F* is the Faraday constant.

On the other hand, knowing the total mass of reacted calcium oxide, the total affected volume *V* can be calculated using the following equation:

(6) $$V=\frac{m_{Ca}}{0.63}\frac{1+w}{\rho },$$

where *ρ* is the density of the cement sample block, which can be obtained experimentally; *w* is the water/cement ratio. The coefficient is 0.63 because the calcium oxide content is about 63% of the OPC binder. Thus, the theoretical volume of the acidification region can be calculated with the following formula:

(7) $$V=k\frac{MIt(w+1)}{0.63\rho nF}.$$

**Figure 11:**
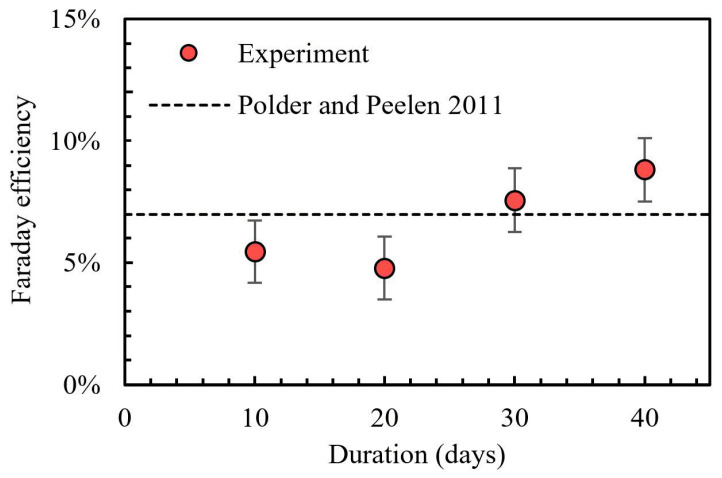
Evolution of the Faraday efficiency of reaction (1) [1].

The Faraday current efficiency is the only unknown parameter in the above equation. A comparison of the experimental results with the empirical estimation given by Polder et al. [1] is shown in Figure 11. According to field records by Polder et al. [1], the Faraday efficiency factor of acid production is estimated to be 7% using a pure empirical method. In our experiment, the Faraday current efficiency is clearly not a constant value. It is lower than 7% in the first period of time, being 5.45% and 4.77% for 10 and 20 days of the experiment. An increase is observed at 30 and 40 days of the experiment. This increase might be attributed to the increase in driven voltage, which accelerates the ionic migration.

The small Faraday current efficiency of reaction (1) does not contradict the fact that it is the dominant anodic reaction. Indeed, the pure acid production by reaction (1) is mitigated by local ionic diffusion and migration to compensate for the loss of hydroxide ions caused by the anodic reaction.

### 4.2. Anodic Acidification-Affected Zone Prediction Using Input Electrical Energy

**Figure 12:**
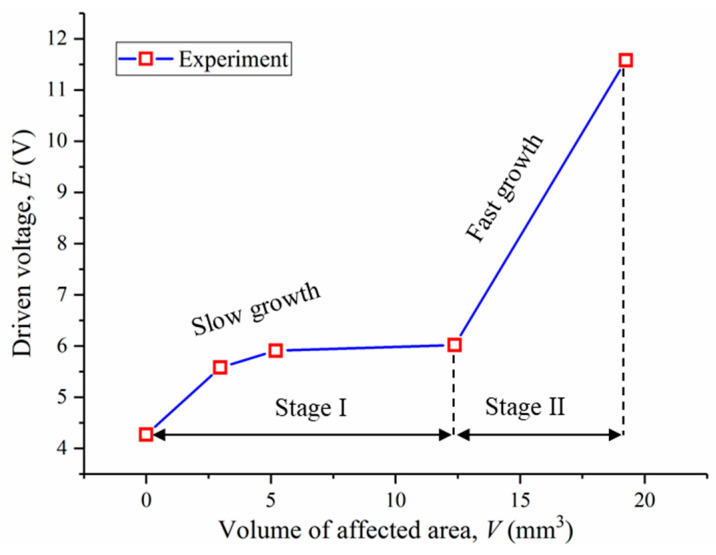
Experimental setup of the accelerated acidification test.

In the literature, it is commonly believed that anodic acidification can cause a significant increase in the electrical resistance of the interface between the primary and secondary anodes, causing a rapid increase in driven voltage. This section studies the correlation between the volume of anodic acidification and the driven voltage. The results are shown in Figure 12, from which it can be seen that two different stages could be identified. The first stage is where the volume of the affected zone is lower than 12.38 mm^3^, which shows a low increase rate of the driven voltage with an increasing volume of the affected zone. The average depth of the affected zone is 0.16 mm. The second stage corresponds to cases where the affected volume is greater than 12.38 mm^3^. A rapid increase in the driven voltage is observed with an increasing volume of the affected zone. This is because when the affected volume is small, it cannot cover the whole interface of the primary and secondary anodes. Therefore, the ions can find a continuous path from the anode to the cathode without bypassing the anodic acidification-affected zone. The results of the μ-XCT analysis for the sample after 40 days of the accelerated ICCP test show that the minor depth of the anodic acidification is 51 μm, while the smallest depth for 30 days is 0 μm, as shown in Figure 9. These results explain the rapid increase in the driven voltage observed at the end of the 36 days of the accelerated ICCP test.

The increase in the driven voltage is also a sign of higher energy consumption. For the purpose of energy saving, the energy consumption of an ICCP system should be as small as possible. However, during the system’s operation period, the energy consumed will increase due to anodic polarization. The input electrical energy is constantly growing along with the total affected volume. The total input electrical energy is defined as follows:

(8) e=∫u(t)i(t)dt,

where *u* is the driven voltage, and *i* is the input current density. Since the input current is kept constant, the input current is hence directly correlated to the cumulative driven voltage as follows:

(9) e=i∫u(t)dt

**Figure 13:**
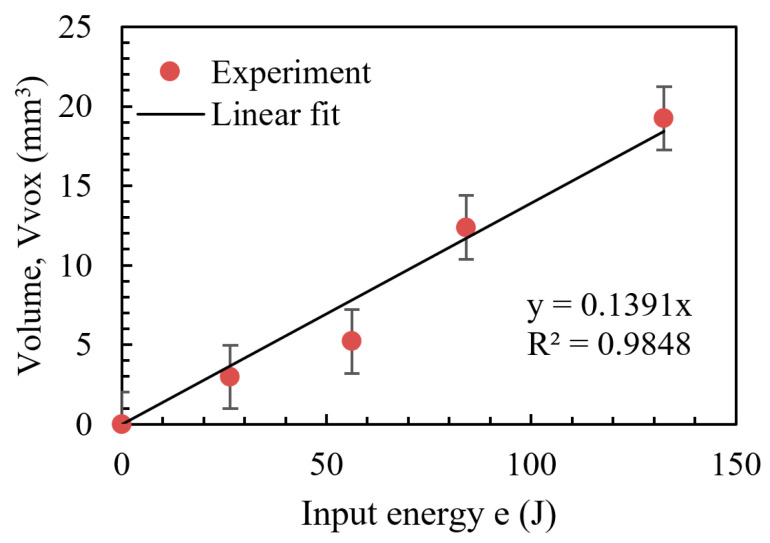
Correlation between the input electrical energy and the affected volume.

The correlation between the input energy and the affected volume *V* is shown in Figure 13, from which it can be seen that the total affected volume is proportional to the input electrical energy. This result indicates that the input electric energy is converted to chemical dissolution energy, the leading cause of the anode acidification. It is possible to decrease the input electrical energy by adding conductive material to the cement paste, such as chopped carbon fibers and graphene [20]. Both carbon fibers and graphene can significantly lower the electrical conductivity of the cement paste, thus decreasing the input electrical energy. Through the above analysis, it can be concluded that the anode acidification rate will also decrease if conductive materials are added. If the anode interface has good conductivity, the voltage in the anode interface region can be significantly reduced, thus reducing the electrochemical reaction rate of the anode interface.

## 5. Conclusions

This article describes a quantitative study of the anodic acidification process using the 3D μ-XCT method. Accelerated acidification tests were conducted using a simulated pore solution, and in situ monitoring of the anodic acidification-affected zone was conducted using 3D μ-XCT. The following conclusions can be drawn:

1. Anodic acidification caused calcium leaching in the cement paste between the primary/secondary interfaces, confirmed by the lower calcium-to-silicon ratio in the EDS spectrum.
2. The acidification-affected zone could be identified as a lower X-ray attenuation zone in μ-XCT images or as a different color in optical microscope analyses.
3. The average Faraday efficiency of the anodic current density was 7.9% at the end of the test, slightly higher than the empirical estimation given by Polder et al. [1]
4. It was found that the volume affected by anodic acidification was proportional to the input electrical energy.

## Figures and Tables

**Table 1:** Chemical composition of PII 42.5 Portland cement used in this study.

Composition	Content (wt.%)
CaO	63.51
SiO_2_	21.86
Al_2_O_3_	4.45
Fe_2_O_3_	2.35
MgO	1.67
K_2_O	0.55
Na_2_O	0.26
SO_3_	2.91
TiO_2_	0.11
LOI *	2.33

* LOI: Loss on ignition.

**Table 2:** Composition of the simulated pore solution according to NACE TM0294-2016 [18].

Composition	Content (wt.%)
Ca(OH)_2_	0.20
KOH	1.00
NaOH	2.45
KCl	3.20
Deionized water	93.15

**Table 3:** The corresponding material phase and the gray level are identified in Figure 6b.

Material	Gray Level
Air	0–78
Acidification-affected zone	79–132
Cement paste	133–181
MMO-coated Ti anode	240–246

**Table 4:** Calculated volume based on segmentation method from the 3D reconstruction.

Duration (Days)	Calculated Volume V (mm^3^)	
	Primary Anode	Affected Zone
0	48.62	0
10	47.92	2.97
20	49.33	5.20
30	48.98	12.38
40	48.06	19.25

## Data Availability

The original contributions presented in the study are included in the article, further inquiries can be directed to the corresponding author.
